# Rural–Urban Differences in the Hypertension Cascade of Care in Northwestern Tanzania: A Community-Based Cross-Sectional Study

**DOI:** 10.21203/rs.3.rs-9645738/v1

**Published:** 2026-06-11

**Authors:** Joshua Elorm Kwawuvi, Bahati Wajanga, Mirlene Perry, Gloria James, Jules Iradukunda, Evarist Msaki, Fortunatus Nestory, Charles Muiruri

**Affiliations:** Duke Global Health Institute; Bugando Medical Centre; Duke University School of Nursing; Bugando Medical Centre; Bugando Medical Centre; Bugando Medical Centre; Bugando Medical Centre; Duke Global Health Institute

**Keywords:** Blood pressure screening, Global health, Hypertension, Hypertension care cascade, Tanzania, Rural–urban disparities

## Abstract

**Background:**

Hypertension is a leading modifiable risk factor for cardiovascular morbidity and mortality worldwide, yet substantial gaps persist in its detection and management in low- and middle-income countries. Understanding where attrition occurs along the hypertension care cascade is critical for identifying high-impact intervention points, particularly in settings undergoing rapid demographic and epidemiological transition.

**Methods:**

The original parent study informing this secondary analysis was a community-based cross-sectional analysis of adults aged 18 years and older in rural and urban districts of Northwestern Tanzania using data from a baseline non-communicable disease survey in 2019. Twelve districts across six regions were selected through a multistage cluster sampling approach. Hypertension care was assessed using a cascade-of-care framework comprising four sequential stages: prior blood pressure screening, prior diagnosis, access to antihypertensive medication, and blood pressure control at the time of screening. Analyses were stratified by rural–urban residence, with statistical comparisons accounting for clustering at the district level.

**Results:**

Among 6,957 participants, 26% reported ever having their blood pressure measured by a health professional, with no significant difference between rural and urban settings (28% vs. 24%, p = 0.09). Among those previously screened, 42% reported a prior diagnosis of hypertension, with a higher proportion among rural residents (45% vs. 39%, p = 0.001). Approximately half of diagnosed individuals reported access to antihypertensive medication, with no rural–urban differences. Among participants with medication access, 64% achieved blood pressure control, with comparable control rates across settings.

**Conclusion:**

The primary bottleneck in hypertension care in Northwestern Tanzania occurs at early detection, with uniformly low screening across rural and urban populations. Once identified and treated, effective blood pressure control is achievable. Strengthening early screening and care activation represents the greatest opportunity to improve hypertension outcomes and provides a critical pre-pandemic baseline to inform policy and programmatic interventions.

## Background

Hypertension is the leading modifiable risk factor for cardiovascular morbidity and mortality worldwide and accounts for more than ten million preventable deaths annually^1,2^. Despite the availability of effective and low-cost treatments, hypertension continues to drive high rates of stroke, heart failure, and premature mortality, largely because it remains undetected and untreated for long periods^3^. The asymptomatic nature of hypertension means that the disease burden is shaped not only by prevalence, but by failures along the continuum of care: from screening and diagnosis to treatment initiation and sustained blood pressure control^4^. Conceptualizing hypertension management as a cascade of care provides a useful framework for identifying where health systems fail to translate disease burden into effective prevention of complications^5^. Low- and middle-income countries (LMICs), including those in sub-Saharan Africa, carry a disproportionate share of the global hypertension burden^4,6^. In these settings, awareness, screening, and treatment coverage remain persistently low, reflecting the historical orientation of the health systems toward acute and infectious conditions rather than the management of chronic diseases ^5,7^. Tanzania exemplifies such a challenge in that hypertension prevalence is high, its complications are a major cause of adult hospitalization and mortality, and yet a substantial proportion of affected individuals remain unaware of their condition^8,9^. While urbanization and health system investment are often assumed to improve detection and management, evidence suggests that gaps in early identification and linkage to care remain widespread across both rural and urban contexts^10^. Northwestern Tanzania presents a crucial setting in which to examine the challenges of hypertension care. The region is undergoing demographic and socioeconomic transition, characterized by population growth and evolving rural–urban structures^11^. Evidence from Northwestern Tanzania indicates a growing burden of hypertension alongside changing age structures and lifestyle-related risk factors^12^. These regional patterns reflect broader national and global trends toward dietary change, reduced physical activity, and increasing exposure to non-traditional foods associated with nutrition and epidemiological transition^13,14^. Historically, rural livelihoods in sub-Saharan Africa were associated with higher physical activity levels and lower cardiometabolic risk, but increasing urbanicity across rural areas is linked with greater prevalence of lifestyle risk factors such as physical inactivity and poor diets, suggesting convergence in rural and urban risk profiles^15^. At the same time, disparities in health system capacity persist, with rural areas relying predominantly on primary-level facilities and lower-cadre health workers, while urban areas host more specialized services^16,17^. Despite these dynamics, there is limited empirical evidence describing how hypertension care pathways differ between rural and urban populations in Northwestern Tanzania, particularly using a cascade-of-care approach. Moreover, few studies provide pre-COVID baseline data against which recent and future changes in hypertension care can be assessed. Understanding these patterns has implications not only for Tanzania but for similar low- and middle-income settings undergoing epidemiological transition. To address this gap, we conducted a large, community-based analysis of the hypertension cascade of care among adults in rural and urban districts of Northwestern Tanzania using data collected in 2019. This study aimed to describe levels of prior blood pressure screening, hypertension diagnosis, access to antihypertensive medication, and blood pressure control, and to compare points of attrition across rural and urban settings.

## Methods

### Study Design and Settings

#### Parent Study Setting

**Figure 1:**
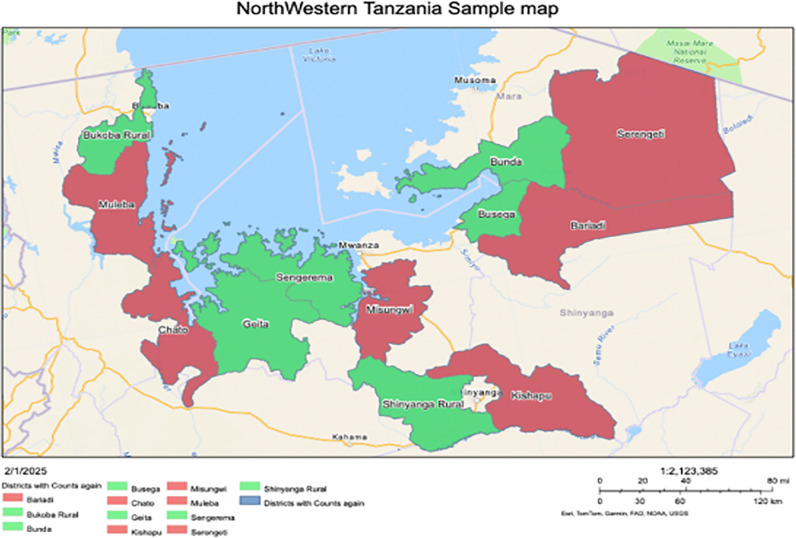
Selected rural and urban districts in Northwestern Tanzania for Screening.

Data for this analysis were drawn from a parent study informing this secondary analysis baseline cross-sectional survey conducted as part of the establishment of a rural–urban non-communicable disease (NCD) cohort in Northwestern Tanzania. The cohort was initiated in November 2019 by Bugando Medical Centre in collaboration with the Catholic University of Health and Allied Sciences (CUHAS).. The baseline survey was designed to assess the burden of major NCDs, including hypertension and Diabetes Mellitus (DM), with the present analysis focused exclusively on hypertension. A multistage cluster sampling approach was employed to select study sites based on administrative boundaries and representative rural–urban comparisons^18^. In the first stage, Northwestern Tanzania was divided into six regions based on administrative boundaries defined by the 2012 Tanzania Population and Housing Census. In the second stage, one rural district and one urban district were purposively selected from each region to ensure representation of both settlement types and to facilitate direct rural–urban comparisons. This process resulted in the selection of twelve districts across the six regions. Rural and urban classifications were based on population size, settlement characteristics, and official designations from the 2012 national census. The selected rural districts were Geita, Bukoba Vijijini, Bunda, Busega, Sengerema, and Shinyanga, while the selected urban districts included Chato, Muleba, Serengeti, Bariadi, Misungwi, and Kishapu, shown in Fig. 1. Northwestern Tanzania is predominantly agrarian, with a largely rural population and a rapidly expanding youth demographic. Within each selected district, a community-based medical screening exercise was conducted to collect health and survey data from adult residents. This design was chosen to allow for regionally distributed rural–urban comparisons of hypertension prevalence and patterns of care rather than to generate population-weighted prevalence estimates for the entire region. This study aimed to assess the hypertension care cascade and compare points of attrition between rural and urban populations in Northwestern Tanzania.

#### Study participants

Eligible participants were adults aged 18 years or older resided in any of the twelve selected districts in Northwestern Tanzania at the time of the screening. Individuals who were acutely ill at the time of data collection or who were cognitively unable to provide informed consent were excluded.. Participants were recruited through community-based and health facility announcements inviting residents to attend NCD screening activities conducted in each district. All individuals who met the eligibility criteria and provided written informed consent were enrolled in the study.

#### Data Collection

Data were collected between March and August 2019 using the World Health Organization (WHO) STEPwise approach to NCD risk factor surveillance (STEPS) questionnaire^19^. The instrument was translated into Kiswahili by a bilingual (English-Kiswahili) team member and adapted to the local context before data collection. A team of trained research staff, working in collaboration with each district’s public health officer, conducted community-based health screening activities in the selected districts. Data collection was conducted using paper-based questionnaires and standardized measurement forms. Completed forms were subsequently entered into REDCap for data management and analysis^20,21^.

#### Health Screening

At each district screening site, eligible participants were individually introduced to the study, provided with detailed information about the procedures, and allowed to ask questions. Written informed consent was obtained before any data collection. Participants who consented were asked to rest for at least 30 minutes before undergoing clinical and anthropometric assessments. Vital signs and anthropometric measurements were collected using standardized procedures recommended in population surveys^22^. Following clinical measurements, participants completed the Kiswahili-translated WHO STEPS questionnaire. Participants who were unable to read or write were assisted by trained research staff to ensure accurate and complete responses. Each screening and survey session lasted approximately 30–45 minutes. Participants identified during screening as having hypotension, defined as a systolic blood pressure < 90 mmHg and/or diastolic blood pressure < 60 mmHg, or hypertensive urgency, defined as systolic blood pressure ≥ 180 mmHg and/or diastolic blood pressure ≥ 110 mmHg, or other medical emergencies were promptly referred to the nearest health facility, typically located within one kilometer of the screening site. Following completion of the screening procedures, participants received brief health education on NCD, including hypertension. A compensation of 10,000 Tanzanian Shillings (equivalent to $US 4.50) was given to all participants at the end of their encounter.

### Definition of Key Variables

#### Blood pressure measurement

Blood pressure was measured by two trained general nurses using a standardized automated digital blood pressure monitor (Omron HEM-907, Tokyo, Japan). Measurements were obtained from the left upper arm with participants seated comfortably, following appropriate cuff size selection and positioning in accordance with standard measurement protocols. Three blood pressure readings were taken at three-minute intervals after participants had rested for at least 30 minutes, and the average of the three readings was used for analysis, consistent with international recommendations for survey-based blood pressure measurement^23^. Participants were classified based on average systolic and diastolic blood pressure values. Uncontrolled hypertension was defined as a mean systolic blood pressure ≥ 140 mmHg and/or a mean diastolic blood pressure ≥ 90 mmHg. Pre-hypertension was defined as a mean systolic blood pressure of 120–139 mmHg and/or a mean diastolic blood pressure of 80–89 mmHg among individuals not meeting criteria for uncontrolled hypertension. Normotension was defined as a mean systolic blood pressure < 120 mmHg and a mean diastolic blood pressure < 80 mmHg^24^.

#### Measurement of Cascade of Care

The hypertension care cascade was operationalized using a series of sequential, self-reported, and measured indicators reflecting key stages of hypertension detection and management. Each stage of the cascade was defined conditionally on the completion of the preceding stage, a standard descriptive epidemiological framework^25^. Blood pressure screening was defined as having ever had one’s blood pressure measured by a health professional, including a nurse, physician, or community health worker, at any point before the study. Among participants who reported prior blood pressure measurement, prior diagnosis of hypertension was defined as self-reported receipt of a hypertension diagnosis by a physician or clinical officer at a health facility. For participants with a prior hypertension diagnosis, access to antihypertensive medication was defined as self-reported availability of prescribed antihypertensive medication within the two weeks preceding the screening. Among participants who reported access to antihypertensive medication, blood pressure control was assessed using blood pressure measurements obtained during the screening. Controlled blood pressure was defined as a mean systolic blood pressure < 140 mmHg and a mean diastolic blood pressure < 90 mmHg, based on the average of three measurements taken at three-minute intervals on the day of screening^26,27^.

### Statistical analyses

All analyses were conducted using R software (version 4.4.0)^28^.Continuous variables were summarized using medians and interquartile ranges (IQRs), while frequencies and proportions were used for categorical variables. Descriptive comparisons of participant characteristics and hypertension-related indicators between rural and urban residents were conducted while accounting for the multistage cluster sampling design. District was treated as the primary clustering unit, reflecting the first stage of sampling. For comparisons of continuous variables between rural and urban groups, cluster-robust t-tests were used to account for intra-cluster correlation within districts. For comparisons of categorical variables, including hypertension status and indicators along the cascade of care, Wald tests were applied with variance estimation adjusted for clustering at the district level. The hypertension cascade of care was analyzed using a descriptive, conditional framework, in which each stage was defined relative to completion of the preceding stage: (1) ever having blood pressure measured, (2) prior diagnosis of hypertension, (3) access to antihypertensive medication, and (4) blood pressure control at the time of screening. Proportions at each stage were calculated overall and stratified by rural–urban residence. Statistical comparisons between rural and urban groups at each stage were conducted using Wald tests with cluster-robust variance estimation. Sampling weights were not applied because the study design was intended to describe and compare patterns of hypertension detection and management between rural and urban settings rather than to estimate population-weighted prevalence or causal associations. All statistical tests were two-sided using a significance level of 0.05.

## Results

### Participant Characteristics

A total of 6,957 participants completed the screening and survey procedures (Table 1). Most participants resided in urban districts (4,134; 59.4%), and 61% were female. The median age was 42 years (IQR: 30–56), with participants in rural areas slightly older than those in urban areas (median 45 vs. 41 years). The majority of participants were married (70%), reported daily physical activity (67%), and had a body mass index (BMI) within the normal range (18.5–24.9 kg/m^2^; 58%). Most participants were self-employed (65%) and reported monthly household earnings below 100,000 Tanzanian shillings (78%). Current smoking status was reported by 6.1% of participants, while 30% reported current alcohol consumption, with a higher prevalence observed in rural areas. Overall, 21% of participants reported awareness of hypertension risk factors, and a small proportion reported a prior diagnosis of diabetes (3%).

### Hypertension Cascade of Care

**Figure 2:**
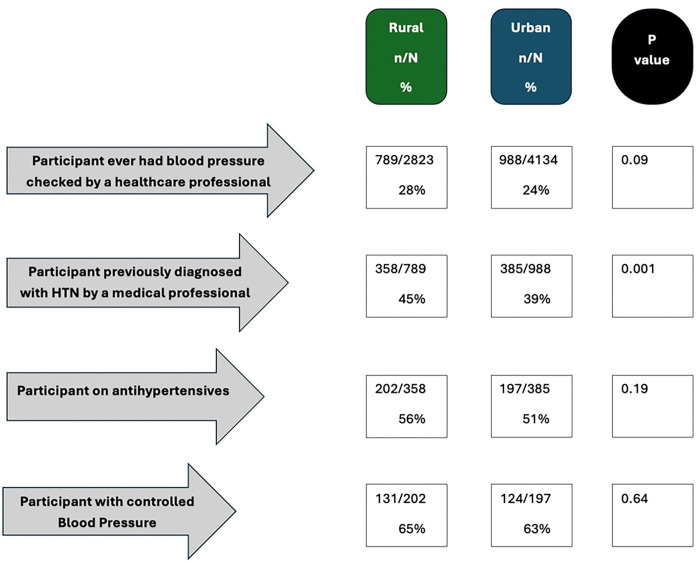
Cascade of Care for Hypertensionbetween Rural and Urban Northwestern Tanzania

Overall, 26% of participants reported having ever had their blood pressure measured by a healthprofessional. There was no statistically significant difference between rural and urban residents in prior blood pressure screening (28% vs. 24%, *p* = 0.09). Among participants previously screened, 42% reporteda prior diagnosis of hypertension by a doctor or clinical officer, with a significantly higher proportionamong rural residents compared with urban residents (45% vs. 39%, *p* = 0.001). For those with a priorhypertension diagnosis, fifty-four percent (54%) reported access to antihypertensive medication withinthe two weeks preceding the survey, with no significant difference between rural and urban settings (*p* = 0.19). Finally, among participants with access to antihypertensive medication, 64% had controlled bloodpressure at the time of screening, with similar control rates observed in rural and urban areas (*p* = 0.64).The hypertension cascade of care is summarized in Table 2 and illustrated in Fig. 2.

## Discussion

In this large, community-based study conducted in Northwestern Tanzania, we identify substantial gaps along the hypertension cascade of care, with the most pronounced breakdown occurring at the earliest stage: blood pressure screening. Fewer than one in three participants reported ever having their blood pressure measured by a health professional, indicating that a large share of individuals with elevated blood pressure in this population likely remain undetected and therefore unlinked to care^29^. From an implementation perspective, failures at this initial step constrain progress across the entire cascade, limiting downstream gains in diagnosis, treatment initiation, and sustained blood pressure control, even where later stages of care appear capable of achieving meaningful clinical outcomes^1,5,29^.

A central finding of this study is the relative similarity of cascade performance between rural and urban settings. Screening levels were low in both contexts, and among individuals who progressed to later stages, access to antihypertensive medication and blood pressure control were largely comparable. This pattern challenges the common assumption that urban residence confers a meaningful advantage in early hypertension detection and management. Rather than indicating equivalent performance across all dimensions of care, these findings suggest that the dominant constraint in this region is not primarily geographic location, but shared, system-level limitations in routine detection and care activation^7,30,31^. In practical terms, the absence of marked rural–urban divergence implies that strengthening a common foundation of screening and linkage mechanisms may be more impactful than pursuing fundamentally different location-specific strategies.

The higher proportion of rural participants reporting a prior hypertension diagnosis among those previously screened warrants cautious interpretation. This difference likely reflects underlying population composition and exposure rather than superior rural diagnostic capacity. Rural participants were older on average and exhibited a higher burden of cardiometabolic risk factors, including higher rates of overweight, tobacco use, and alcohol consumption, as well as lower socioeconomic status, all of which increase the likelihood of diagnosis upon interaction with the health system^32^. At the same time, Northwestern Tanzania is undergoing demographic and lifestyle transitions that may be narrowing historic rural–urban differences in risk profiles. As patterns of work, transport, diet, and alcohol use evolve across both settings, traditional distinctions between “rural” and “urban” cardiometabolic risk environments become less distinct, reinforcing the need for broadly applicable detection strategies^30,33^.

Despite substantial losses early in the cascade, a more encouraging pattern emerged at later stages. Approximately half of the participants with a prior hypertension diagnosis reported access to antihypertensive medication, and among those with access, nearly two-thirds had controlled blood pressure at the time of screening. This finding is critical: once individuals are identified and linked to treatment, effective blood pressure control appears achievable in both rural and urban settings^34,35^. These results suggest that the primary barrier to improved hypertension outcomes in this context is not treatment effectiveness or adherence alone, but failure to detect hypertension and initiate care for a large proportion of affected individuals.

Taken together, these findings point to a clear programmatic implication. The greatest gains in hypertension control in Northwestern Tanzania are likely to come from strengthening early screening and care activation rather than developing separate rural and urban treatment approaches. A unified strategy that expands routine blood pressure measurement across healthcare encounters, increases community-based screening opportunities, and improves follow-up after initial detection can be deployed across settings while allowing for context-specific adaptations in delivery. Importantly, this approach avoids reinforcing an artificial rural–urban divide in program design where the data suggest that early cascade failures are broadly shared.

Finally, this analysis provides an important pre-COVID baseline for hypertension care in Northwestern Tanzania. Establishing cascade performance before the pandemic clarifies where the health system was already underperforming and creates a reference point against which subsequent disruptions or improvements can be evaluated. Future post-COVID analyses can build on this baseline to assess whether observed changes reflect deterioration, recovery, or meaningful progress in hypertension detection and management. In all, this study reframes the core hypertension challenge in the region as one of early detection and care activation rather than treatment efficacy, highlighting where policy and programmatic investments are likely to yield the greatest impact.

### Strengths and limitations

This study has several limitations that should be considered when interpreting the findings. First, information on prior blood pressure measurement, hypertension diagnosis, and access to antihypertensive medication was self-reported and may be subject to recall or social desirability bias. Second, the cross-sectional design limits causal inference regarding observed rural–urban differences and associated factors. Third, the multistage sampling strategy was intended to facilitate rural–urban comparisons rather than to generate population-weighted prevalence estimates for the entire region. Finally, data were collected in 2019, before the COVID-19 pandemic, which has since disrupted health services and may have further affected hypertension detection and management^36^. Despite these limitations, this study provides valuable pre-COVID baseline evidence on gaps along the hypertension care cascade in Northwestern Tanzania. By explicitly examining points of attrition from screening through control, the findings highlight missed opportunities for early detection and underscore the potential for improved outcomes through strengthened screening and linkage to care. As Tanzania and similar settings continue to undergo rapid demographic and epidemiological transitions, addressing early cascade failures will be critical to reducing downstream cardiovascular morbidity and mortality.

## Conclusion

This study demonstrates that the principal bottleneck in hypertension care in Northwestern Tanzania lies at the stage of early detection, with persistently low blood pressure screening rates across both rural and urban populations. Contrary to common assumptions of an urban advantage in hypertension management, performance along the care cascade was largely similar between settings. Importantly, once individuals were diagnosed and had access to antihypertensive medication, blood pressure control was relatively high, indicating that effective hypertension management is achievable when patients are successfully linked to care. These findings suggest that the greatest gains in hypertension control in this region are likely to come from strengthening early screening and care activation rather than from developing separate rural and urban treatment strategies. Expanding routine and community-based blood pressure screening, improving follow-up after initial detection, and ensuring timely linkage to diagnosis and treatment represent the most impactful intervention points. As a pre-COVID baseline, this study provides critical evidence to inform future policy and programmatic efforts aimed at reducing hypertension-related morbidity and mortality across Northwestern Tanzania.

## Figures and Tables

**Table 1:** Characteristics of Study Participants

Variable.	Overall N = 6957	Rural 2823 (40.6%)	Urban 4134 (59.4%)
**Sex**			
Male	2713(39%)	1086(38%)	1627(39%)
Female	4244(61%)	1737(62%)	2507(61%)
**Age**	42(30–56)	45(32–57.5)	41(28–55)
**Married**	4867(70%)	2083(74%)	2784(67%)
**BMI**	23.3(20.93–26.78)	23.24(20.76–26.72)	23.36(20.96–26.78)
**BMI ranges**			
< 18.5	456(6.6%)	234(8.3%)	222(5.4%)
18.5 - (< 25)	4052(58%)	1593(56%)	2459(59%)
25 - (30)	1547(22%)	601(21%)	946(23%)
> 30	902(13%)	395 (14%)	507 (12%)
**Smoking**	425 (6.1%)	228(8.1%)	197(4.8%)
**Alcohol**	2068(30%)	959(34%)	1109(27%)
**Aware of risk factors of hypertension**	1435(21%)	523(19%)	912(22%)
**History of Diabetes**	179 (3%)	90 (3%)	89 (2%)
**Moderate-intensity physical activity**	4656(67%)	2053(73%)	2603(63%)
**Average household monthly earnings (TSH)**			
< 20000	2705(40%)	1238(44%)	1557(38%)
20,000-100000	2647(38%)	1014(36%)	1633(40%)
100000–500000	1258(18%)	492(17%)	766(19%)
> 500000	257(3.7%)	79(2.8%)	178(4.3%)

**Table 2:** Differences in Cascade of Care for Hypertension between Rural and Urban Northwestern Tanzania

Variable	Overall (n, N) %	Rural 2823, 40.6%	Urban 4134, 59.4%	P value
**Number of participants who have had their blood pressure checked**	1777/6957, (26%)	789/2823, (28%)	988/4134, (24%)	0.09
**Number of participants with previous diagnosis of hypertension**	743/1777, (42%)	358/789, (45%)	385/988, (39%)	**0.001**
**Number of participants who are on medication**	399/743, (54%)	202/358, (56%)	197/385, (51%)	0.19
**Number of participants with blood pressure under control**	255/399, (64%)	131/202, (65%)	124/197, (63%)	0.64

## Data Availability

The datasets generated and/or analyzed during the current study are not publicly available due to ethical restrictions related to participant confidentiality and consent. De-identified data may be made available from the corresponding author on reasonable request, subject to approval by the Duke University Health System Institutional Review Board and the Bugando Medical Centre Institutional Review Board.

## Abbreviations

BMI: Body Mass Index
CUHAS: Catholic University of Health and Allied Sciences
DM: Diabetes Mellitus
IQR: Interquartile Range
LMICs: Low- and Middle-Income Countries
NCDs: Non-Communicable Diseases
REDCap: Research Electronic Data Capture
STEPS: STEPwise Approach to Non-Communicable Disease Risk Factor Surveillance
WHO: World Health Organization

## References

[1] Mills KT, Stefanescu A, He J. The global epidemiology of hypertension. Nat Rev Nephrol. 2020 Apr;16(4):223–37. doi:10.1038/s41581-019-0244-2 32024986 PMC7998524

[2] Murray CJL, Aravkin AY, Zheng P, Abbafati C, Abbas KM, Abbasi-Kangevari M, Global burden of 87 risk factors in 204 countries and territories, 1990–2019: a systematic analysis for the Global Burden of Disease Study 2019. The Lancet. 2020 Oct 17;396(10258):1223–49. doi:10.1016/S0140-6736(20)30752-2 PMC7566194 33069327

[3] Forouzanfar MH, Liu P, Roth GA, Ng M, Biryukov S, Marczak L, Global Burden of Hypertension and Systolic Blood Pressure of at Least 110 to 115 mm Hg, 1990–2015. JAMA. 2017 Jan 10;317(2):165–82. doi:10.1001/jama.2016.19043 28097354

[4] Mills KT, Bundy JD, Kelly TN, Reed JE, Kearney PM, Reynolds K, Global Disparities of Hypertension Prevalence and Control: A Systematic Analysis of Population-Based Studies From 90 Countries. Circulation. 2016 Aug 9;134(6):441–50. doi:10.1161/CIRCULATIONAHA.115.018912 27502908 PMC4979614

[5] Geldsetzer P, Manne-Goehler J, Marcus ME, Ebert C, Zhumadilov Z, Wesseh CS, The state of hypertension care in 44 low-income and middle-income countries: a cross-sectional study of nationally representative individual-level data from 1·1 million adults. Lancet Lond Engl. 2019 Aug 24;394(10199):652–62. doi:10.1016/S0140-6736(19)30955-9 31327566

[6] Zhou B, Perel P, Mensah GA, Ezzati M. Global epidemiology, health burden and effective interventions for elevated blood pressure and hypertension. Nat Rev Cardiol. 2021 Nov;18(11):785–802. doi:10.1038/s41569-021-00559-8 34050340 PMC8162166

[7] Ataklte F, Erqou S, Kaptoge S, Taye B, Echouffo-Tcheugui JB, Kengne AP. Burden of undiagnosed hypertension in sub-saharan Africa: a systematic review and meta-analysis. Hypertens Dallas Tex 1979. 2015 Feb;65(2):291–8. doi:10.1161/HYPERTENSIONAHA.114.04394 25385758

[8] Peck RN, Green E, Mtabaji J, Majinge C, Smart LR, Downs JA, Hypertension-related diseases as a common cause of hospital mortality in Tanzania: a 3-year prospective study. J Hypertens. 2013 Sep;31(9):1806–11. doi:10.1097/HJH.0b013e328362bad7 23777761 PMC4005815

[9] Zhou B, Bentham J, Cesare MD, Bixby H, Danaei G, Cowan MJ, Worldwide trends in blood pressure from 1975 to 2015: a pooled analysis of 1479 population-based measurement studies with 19·1 million participants. The Lancet. 2017 Jan 7;389(10064):37–55. doi:10.1016/S0140-6736(16)31919-5 PMC5220163 27863813

[10] Manne-Goehler J, Atun R, Stokes A, Goehler A, Houinato D, Houehanou C, Diabetes diagnosis and care in sub-Saharan Africa: pooled analysis of individual data from 12 countries. Lancet Diabetes Endocrinol. 2016 Nov;4(11):903–12. doi:10.1016/S2213-8587(16)30181-4 27727123

[11] datadot [Internet]. [cited 2024 Dec 12]. United Republic of Tanzania. Available from: https://data.who.int/countries/834

[12] Mosha NR, Mahande M, Juma A, Mboya I, Peck R, Urassa M, Prevalence,awareness and factors associated with hypertension in North West Tanzania. Glob Health Action. 2017 Jun 9;10(1):1321279. doi:10.1080/16549716.2017.1321279 28598724 PMC5496079

[13] Keding GB, Msuya JM, Maass BL, Krawinkel MB. Dietary Patterns and Nutritional Health of Women: The Nutrition Transition in Rural Tanzania. Food Nutr Bull. 2011 Sep 1;32(3):218–26. doi:10.1177/156482651103200306 22073796

[14] Popkin BM, Adair LS, Ng SW. Global nutrition transition and the pandemic of obesity in developing countries. Nutr Rev. 2012 Jan 1;70(1):3–21. doi:10.1111/j.1753-4887.2011.00456.x 22221213 PMC3257829

[15] Riha J, Karabarinde A, Ssenyomo G, Allender S, Asiki G, Kamali A, Urbanicity and Lifestyle Risk Factors for Cardiometabolic Diseases in Rural Uganda: A Cross-Sectional Study. PLOS Med. 2014 Jul 29;11(7):e1001683. doi:10.1371/journal.pmed.1001683 25072243 PMC4114555

[16] Munga MA, Mæstad O. Measuring inequalities in the distribution of health workers: the case of Tanzania. Hum Resour Health. 2009 Jan 21;7(1):4. doi:10.1186/1478-4491-7-4 19159443 PMC2655278

[17] Shemdoe A, Mbaruku G, Dillip A, Bradley S, William J, Wason D, Explaining retention of healthcare workers in Tanzania: moving on, coming to ‘look, see and go’, or stay? Hum Resour Health. 2016 Jan 19;14(1):2. doi:10.1186/s12960-016-0098-7 26783192 PMC4717661

[18] STEPwise approach to NCD risk factor surveillance (STEPS) [Internet]. [cited 2026 Jan 23]. Available from: https://www.who.int/teams/noncommunicable-diseases/surveillance/systems-tools/steps

[19] Riley L, Guthold R, Cowan M, Savin S, Bhatti L, Armstrong T, The World Health Organization STEPwise Approach to Noncommunicable Disease Risk-Factor Surveillance: Methods, Challenges, and Opportunities. Am J Public Health. 2016 Jan;106(1):74–8. doi:10.2105/AJPH.2015.302962 26696288 PMC4695948

[20] Harris PA, Taylor R, Thielke R, Payne J, Gonzalez N, Conde JG. Research electronic data capture (REDCap)--a metadata-driven methodology and workflow process for providing translational research informatics support. J Biomed Inform. 2009 Apr;42(2):377–81. doi:10.1016/j.jbi.2008.08.010 18929686 PMC2700030

[21] Harris PA, Taylor R, Minor BL, Elliott V, Fernandez M, O’Neal L, The REDCap consortium: Building an international community of software platform partners. J Biomed Inform. 2019 Jul;95:103208. doi:10.1016/j.jbi.2019.103208 31078660 PMC7254481

[22] Manual [Internet]. [cited 2026 Jan 23]. Available from: https://www.who.int/teams/noncommunicable-diseases/surveillance/systems-tools/steps/manuals

[23] Firima E, Retselisitsoe L, Leisa I, Manthabiseng M, Sematle MP, Bane M, Head-to-head comparison of the WHO STEPwise approach with immediate unattended and delayed unattended automated blood pressure measurements during household-based screening: a diagnostic accuracy study in Lesotho. eClinicalMedicine. 2023 Sep 1;63:102197. doi:10.1016/j.eclinm.2023.102197 37680951 PMC10480531

[24] Chobanian AV, Bakris GL, Black HR, Cushman WC, Green LA, Izzo JL, Seventh report of the Joint National Committee on Prevention, Detection, Evaluation, and Treatment of High Blood Pressure. Hypertension. 2003 Dec;42(6):1206–52. doi:10.1161/01.HYP.0000107251.49515.c2 14656957

[25] Jobe M, Mactaggart I, Hydara A, Kim MJ, Bell S, Badjie O, Evaluating the hypertension care cascade in middle-aged and older adults in The Gambia: findings from a nationwide survey. eClinicalMedicine. 2023 Oct 1;64. doi:10.1016/j.eclinm.2023.102226 PMC10520336 37767194

[26] Crim MT, Yoon SS (Sarah), Ortiz E, Wall HK, Schober S, Gillespie C, NATIONAL SURVEILLANCE DEFINITIONS FOR HYPERTENSION PREVALENCE AND CONTROL AMONG ADULTS. Circ Cardiovasc Qual Outcomes. 2012 May;5(3):343–51. doi:10.1161/CIRCOUTCOMES.111.963439 22550130 PMC3407684

[27] Mousavi SS, Reyna MA, Clifford GD, Sameni R. A Survey on Blood Pressure Measurement Technologies: Addressing Potential Sources of Bias. Sensors. 2024 Mar 6;24(6). doi:10.3390/s24061730 PMC10976157 38543993

[28] Framework for Easy Statistical Modeling, Visualization, and Reporting [Internet]. [cited 2026 Mar 28]. Available from: https://easystats.github.io/easystats/

[29] Osetinsky B, Mhalu G, Mtenga S, Tediosi F. Care cascades for hypertension and diabetes: Cross-sectional evaluation of rural districts in Tanzania. PLoS Med. 2022 Dec;19(12):e1004140. doi:10.1371/journal.pmed.1004140 36469527 PMC9762578

[30] Gafane-Matemane LF, Craig A, Kruger R, Alaofin OS, Ware LJ, Jones ESW, Hypertension in sub-Saharan Africa: the current profile, recent advances, gaps, and priorities. J Hum Hypertens. 2024 May 2;1–16. doi:10.1038/s41371-024-00913-6 PMC11867975 38698111

[31] Guwatudde D, Nankya-Mutyoba J, Kalyesubula R, Laurence C, Adebamowo C, Ajayi I, The burden of hypertension in sub-Saharan Africa: a four-country cross sectional study. BMC Public Health. 2015 Dec 5;15:1211. doi:10.1186/s12889-015-2546-z 26637309 PMC4670543

[32] Hypertension [Internet]. [cited 2026 Jan 23]. Available from: https://www.who.int/news-room/fact-sheets/detail/hypertension

[33] Dalal S, Beunza JJ, Volmink J, Adebamowo C, Bajunirwe F, Njelekela M, Non-communicable diseases in sub-Saharan Africa: what we know now. Int J Epidemiol. 2011 Aug;40(4):885–901. doi:10.1093/ije/dyr050 21527446

[34] Gupta R, Gaur K, S Ram CV. Emerging trends in hypertension epidemiology in India. J Hum Hypertens. 2019 Aug;33(8):575–87. doi:10.1038/s41371-018-0117-3 30254382

[35] Lamloum D, Fassio F, Osetinsky B, Tediosi F. Care Cascades for Hypertension in Low-Income Settings: A Systematic Review and Meta-Analysis. Int J Public Health. 2023 Oct 12;68:1606428. doi:10.3389/ijph.2023.1606428 37901590 PMC10600349

[36] The impact of the COVID-19 pandemic on noncommunicable disease resources and services: results of a rapid assessment [Internet]. [cited 2026 Jan 23]. Available from: https://www.who.int/publications/i/item/9789240010291. Accessed 23 Jan 2026.

